# Probiotics for oral health: a critical evaluation of bacterial strains

**DOI:** 10.3389/fmicb.2024.1430810

**Published:** 2024-06-24

**Authors:** Rachelle E. Beattie

**Affiliations:** ProBiora Health, LLC, Sarasota, FL, United States

**Keywords:** oral care, probiotic, prebiotic, *Streptococcus*, *Lactobacillus*, dentistry

## Abstract

Oral health is critical for total body health and well-being; however, little improvement in oral health status has occurred in the U.S. over the past 20 years. Tooth decay and gum disease remain highly prevalent, with more than 90% and 50% of adults suffering from these conditions, respectively. To combat this lack of improvement, alternative approaches to dental care are now being suggested. One such alternative therapy is probiotics for oral care. In the oral cavity, probiotic strains have been shown to reduce levels of oral pathogens, inhibit the formation of dental caries, and reduce the levels of bacteria that cause halitosis. However, as the oral care probiotic market expands, many products contain bacterial species and strains with no documented health benefits leading to confusion and mistrust among consumers and clinicians. This confusion is enhanced by the regulatory status of probiotic products which puts the onus of safety and efficacy on the manufacturer rather than a central regulatory body. The overarching goal of this review is to provide consumers and clinicians with documented evidence supporting (or refuting) the health benefits of oral care probiotics marketed for sale in the United States. This includes defining what constitutes an oral care probiotic product and a strain level analysis of candidate probiotics from the genera *Streptococcus, Lactobacillus, Bifidobacterium,* and *Bacillus.* Additionally, prebiotics and postbiotics will be discussed. Finally, a set of considerations for consumers and clinicians is provided to empower probiotic product decision making. Together, this review will improve understanding of oral care probiotics marketed in the US for dental professionals and consumers.

## 1 Introduction

Despite technological and medical advancements, the oral health of American adults has not improved significantly during the past 20 years (National Institute of Dental and Craniofacial Research, 2022). Gum disease and tooth decay continue to affect a majority of the population, with 52% of children diagnosed with at least one cavity by the age of 8 (Centers for Disease Control and Prevention, 2021). Poor oral health is linked to an array of systemic health problems including diabetes, heart disease, and dementia (Centers for Disease Control and Prevention, 2021; National Institute of Dental and Craniofacial Research, 2022). Oral health conditions including cavities, gum disease, and tooth loss also directly affect quality of life, influencing social interactions, employment opportunities, and self-confidence (Kaur et al., 2017; National Institute of Dental and Craniofacial Research, 2022).

Historically, dental care has focused on three preventative therapies: tooth brushing, flossing, and fluoride treatment. However, these recommended therapies have not changed since the introduction of fluoride in drinking water in 1945 (Unde et al., 2018). When preventative methods fail, oral health conditions are treated using reactive therapy, often applied in advanced stages of disease progression (Nock, 2024). Methods including scaling and root planing, fillings, and antibiotic application (among others) have been used in reactive dentistry for more than 50 years (Yilmaz et al., 1994), but these methods have had limited success improving the overall status of oral health in the United States (National Institute of Dental and Craniofacial Research, 2022). Due to the lack of success, alternative proactive approaches to dental care are needed to improve oral health. One such alternative therapy is probiotics.

In 2001, the term “probiotic” was formally defined by the World Health Organization as, “live microorganisms which when administered in adequate amounts confer a health benefit on the host” (Food and Agriculture Organization and World Health Organization Expert Consultation, 2001). Probiotics have primarily been marketed to the general public for gastrointestinal disease therapy and, to date, most consumers affiliate probiotic bacteria with gut health. However, the oral cavity is the ideal environment for the application of probiotic therapy because many of the diseases that affect the mouth originate from dysbiosis of the oral microbiome. For example, the primary cause of periodontitis is an increase in key dental pathogens such as *Porphyromonas gingivalis* (How et al., 2016; Abdulkareem et al., 2023). In a healthy oral cavity, *P. gingivalis* may be present in the dental biofilm at very low concentrations, but stressors including diet, lifestyle, and individual susceptibility can increase the abundance of this pathogen resulting in disease (How et al., 2016). Cavities also originate from oral dysbiosis, as the primary etiological agent is an overabundance of *Streptococcus mutans* (Forssten et al., 2010). Other oral conditions, such as halitosis (or bad breath) are also associated with microbial dysbiosis. Individuals suffering from halitosis often have an overabundance of microorganisms that produce volatile sulfur compounds compared to those without halitosis (Haraszthy et al., 2007).

The concept of bacterial probiotic therapy for oral health was first reported in 1985 when Hillman et al. isolated multiple strains of *Streptococcus* spp. from healthy subgingival plaque. These bacterial strains were capable of inhibiting the growth of periodontal pathogens including *Fusobacterium nucleatum*, *Aggregatibacter actinomycetemcomitans*, and *Porphyromonas gingivalis* (previously *Bacteroides gingivalis*; Hillman et al., 1985). The method of inhibition was shown to be hydrogen peroxide production, a natural metabolic biproduct of the isolated *Streptococcus* strains (Hillman et al., 1985). Hillman’s foundational work led to the concept of replacement therapy in the oral cavity. Replacement therapy maintains balance in the oral microbiome by replacing disease causing microorganisms with a higher abundance of beneficial microorganisms through competitive exclusion (Hillman et al., 1987, 2009). This concept continues to serve as the basis for the selection of probiotics for oral health therapy today.

The demand for proactive therapy continues to increase across the dental landscape, resulting in an exponential expansion of the oral-care probiotic market in the United States. The first probiotic product specifically designed for oral-care was marketed in the mid-2000s. Today, more than 25 companies market probiotic products for oral health with over 50 bacterial species and strains included across the products (How and Yeo, 2021). Many of these products contain bacterial species or strains that are “Generally Recognized as Safe” (GRAS), or available for inclusion in food products and dietary supplements based on a history of safe use. However, safety does not necessitate efficacy, and many bacterial strains used in oral care probiotics have no documented health benefit in the oral cavity (Van Holm et al., 2023).

As the market grows, dental professionals and consumers may be overwhelmed by the probiotic products available for use. Conflicting, misleading, or confusing information supplied by competing product manufacturers can overshadow peer-reviewed research and may lead to public distrust of probiotics as a proactive dental therapy. The overarching goal of this review is to demystify the US oral-care probiotic market by providing an in-depth analysis of the science behind bacterial strains currently included in oral-care probiotics. Critical details including the origin of isolation, documented probiotic benefit (s) in the oral cavity (or lack thereof), dosage, efficacy, and safety of the probiotic strains is included. Prebiotics, postbiotics, and the state of the oral health probiotics market is also reviewed. Together, this information will help dental professionals and consumers understand both the science supporting the use of oral-care probiotics and how to sift through marketing messages for a product that delivers targeted, research-backed health benefits.

## 2 Defining effective probiotics for oral care

Developing a probiotic requires careful consideration of multiple factors including (but not limited to): the isolation location of the bacterial strain, ability to survive in the desired body area, safety of the strain, efficacy in the desired body area, ability to ferment on an industrial scale, and potential contraindications in other body areas. Here, the steps used to develop a probiotic product in the United States are outlined, but many of the principles apply globally. The first step for a targeted probiotic is to define the area in which the probiotic effect should occur. For an oral care probiotic, health effects should be expected in the regions covered by the oral cavity, which is defined by the National Institutes of Health as, “*refers to the mouth…it includes the lips, the lining inside the cheeks and lips, the front two thirds of the tongue, the upper and lower gums, the floor of the mouth under the tongue, the bony roof of the mouth, and the small area behind the wisdom teeth”* (National Cancer Institute, 2024). This area also houses the teeth.

**Figure 1:**
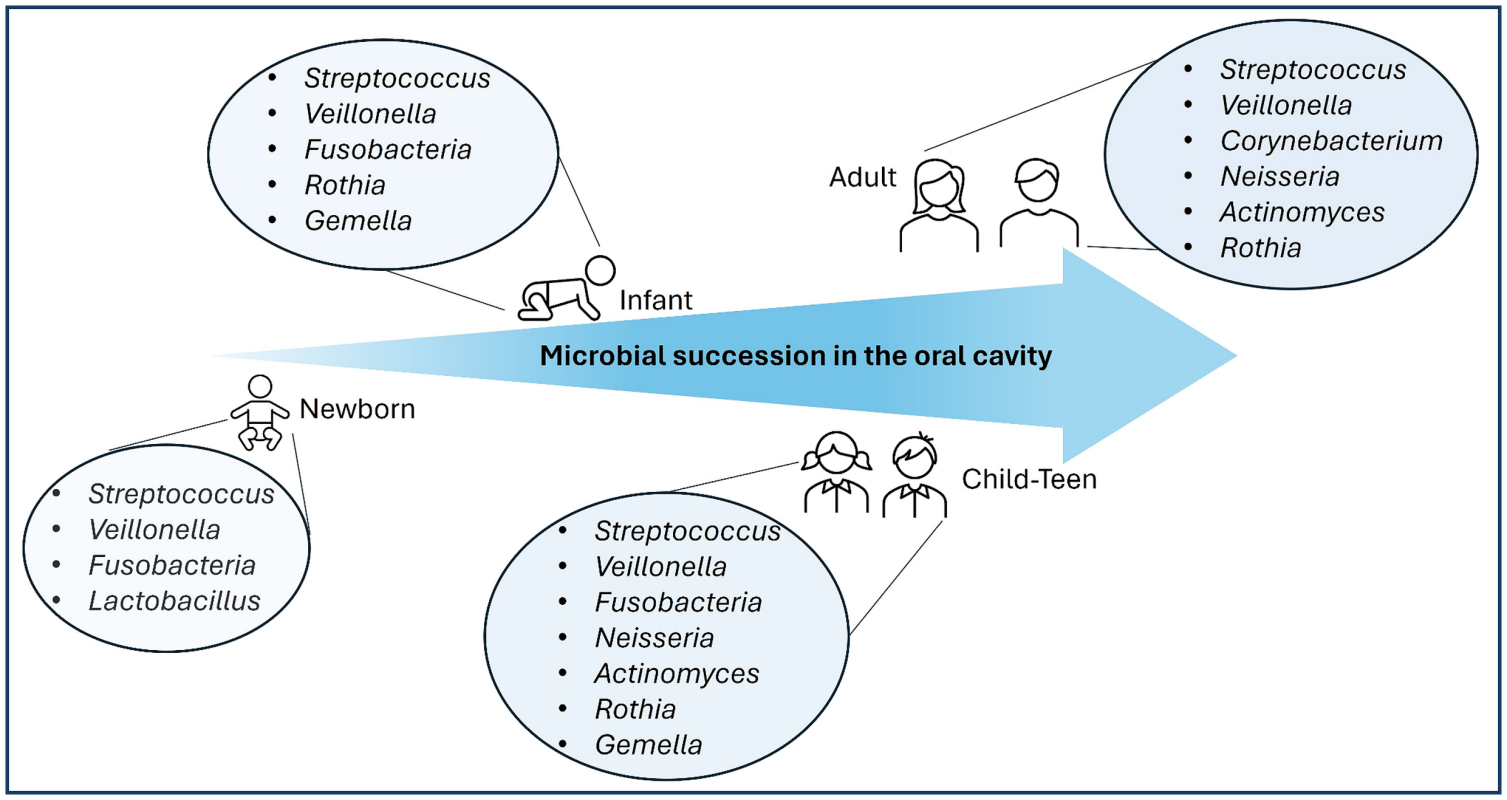
Microbial succession of predominant genera in a healthy oral cavity.

Next, one must consider the location of isolation. Logically, one would propose that a probiotic for oral care should be isolated from the oral cavity; however, the oral cavity contains over 700 species of bacteria (Aas et al., 2005). To narrow down possible probiotic candidates, bacteria routinely found in healthy mouths should be considered first. Sequencing technologies have made this considerably simpler. Bacterial succession from infancy through adulthood follows a common trajectory in a healthy oral cavity. In the first few days of life, members of the genera *Streptococcus*, *Veillonella*, and *Fusobacteria* serve as early colonizers (Dzidic et al., 2018). Members of the genera *Rothia* and *Gemella* arrive before the age of one, followed by late colonizers including members of *Neisseria* and *Actinomyces* arriving after one year of age (Dzidic et al., 2018; Figure 1). In a healthy adult oral cavity, members of the genera *Streptococcus, Rothia, Neisseria, Veillonella,* and *Actinomyces* dominate (Deo and Deshmukh, 2019) while pathogenic species of the genera *Tannerella, Bifidobacterium*, and *Prophyromonas* were found in diseased mouths (Aas et al., 2005; Willis and Gabaldón, 2020; Figure 1). While sequencing information can help identify species routinely found in healthy mouths, an estimated 40%–60% of the bacteria in the oral cavity cannot be grown in the lab (Siqueira and Rôças, 2013), further limiting potential probiotic strains. Bacterial strains found in healthy mouths and capable of growth outside the oral cavity move on to the next stage of evaluation to be considered for use as a probiotic.

Following the selection steps above, safety and efficacy of the probiotic strain candidates must be evaluated. Bacteria, as a group, are generally beneficial organisms contributing a wide range of functions essential for life on earth. While only a small proportion of bacteria cause infection and disease (Doron and Gorbach, 2008), serious illness and even death can occur if probiotic candidates are not thoroughly vetted. Extreme caution must be used to verify that bacterial strains considered for probiotic applications are safe for human use, non-pathogenic, and are resistant to at least a portion of commonly available antibiotics (Sanders et al., 2010; Pradhan et al., 2020). Verification of these factors has become markedly simple with advances in sequencing technologies. Entire bacterial genomes, proteomes, and even resistomes can be sequenced and evaluated for relatively low cost at high resolution (Satam et al., 2023). Whole genome sequencing, virulence assessments, antibiotic resistance evaluations, and genetic stability should be the minimum standard for new probiotic strains being considered for the market.

Once a probiotic candidate strain has been isolated from the desired body location, found capable of growing in the laboratory, and deemed safe for use, a thorough analysis of efficacy should be conducted. For oral care probiotics, one must consider what health benefit (s) are desired and can be achieved by a bacterial strain. Probiotics exert health benefits through a variety of mechanisms including competitive exclusion, antimicrobial compound production, bacteriocin production, immune modulation, and interactions with the host endocrine system (Bermudez-Brito et al., 2012; Plaza-Diaz et al., 2019). In the oral cavity, many disease states are the direct result of overgrowth of pathogenic bacteria. Gingivitis, periodontitis, and caries have etiologies of bacterial or microbial origin (Tatakis and Kumar, 2005; Chen et al., 2020); thus, probiotic bacteria that inhibit the growth of or complete for attachment sites with oral pathogens are prime candidates for oral care probiotics. In addition to identifying the potential health benefits of a probiotic candidate strain, a review of interactions between the strain and other oral microorganisms is critical. For example, multiple lactic acid bacteria of the genus *Lactobacillus* have been proposed as oral care probiotics citing reduced inflammation of oral tissues following use. However, *Lactobacillus* spp. have been implicated in the formation and progression of dental caries (Caufield et al., 2015; Shimada et al., 2015), suggesting the risk outweighs the potential benefit. This example, explored in more detail in Section 4.2. below, highlights the need for a thorough review of the probiotic interactions within the oral cavity in addition to the mechanism of action.

Following safety and efficacy assessments, marketability and scalability of the probiotic candidate strain must be assessed. Probiotic products sold to consumers must meet multiple manufacturing and consumer requirements that are rarely considered during the strain isolation stage. Manufacturing considerations including strain yield, Good Manufacturing Practices (GMP), absence of contaminants, product consistency, and product stability across varying temperatures and humidity are critical to a successful probiotic product (Fenster et al., 2019) while consumer preferences may include shelf-life, ease-of-use, and sustainability (Siddiqui et al., 2023). Additionally, probiotic candidate strains may be patented for specific uses or in specific combinations, which may limit the use of the probiotic candidate strain (s) to individual companies or designated dosage forms. Businesses considering using probiotic strains in their products must evaluate the strain for propensity to produce a high-yield (>100 Billion colony forming units/g) in an industrial fermentation setting which is not possible for all probiotic candidates (Fenster et al., 2019). Additionally, probiotic stability factors including shelf-life, resistance to contamination, and probiotic viability, are critical for marketed probiotic products.

**Figure 2:**
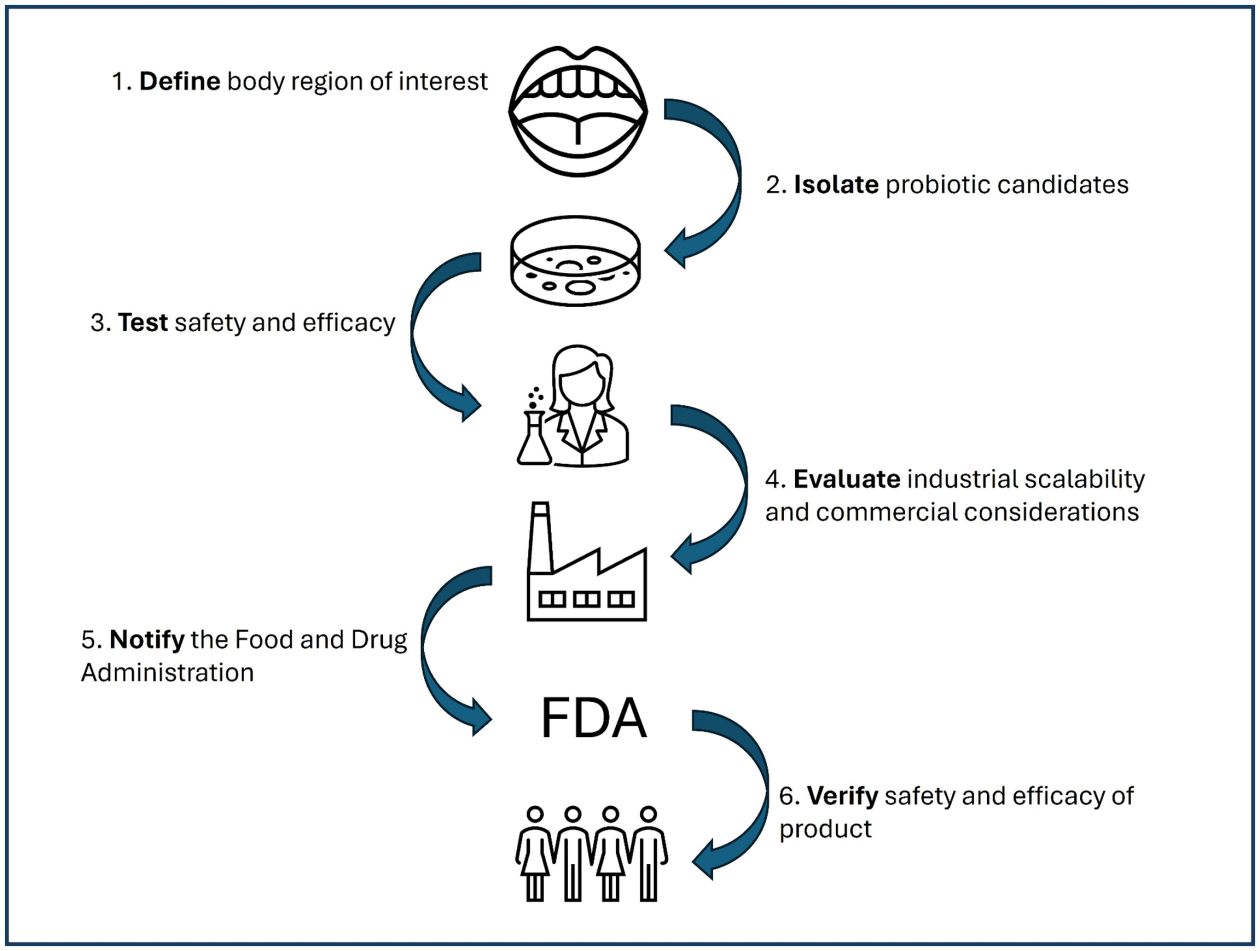
Overview of the probiotic product development process from candidate strain isolation through consumer verification of safety and efficacy.

Probiotic candidate strains that are shown to be safe, effective, and scalable are considered ready for the USA market. In the USA, most probiotic products are marketed as dietary supplements or functional food ingredients which only require pre-market notification, not approval, by the U.S. Food and Drug Administration (FDA). Of special note, the FDA does not approve dietary supplements or functional food ingredients for their safety and efficacy prior to sale; it is considered the responsibility of the manufacturer to ensure the products are safe and labeled following FDA guidelines (United States Food and Drug Administration, 2023). However, new dietary ingredients, or those that that were not marketed in a dietary supplement or as functional food ingredients before 15 October 1994, must notify the FDA at least 75 days prior to sale (U.S. Department of Health and Human Services, 2024). New dietary ingredients include newly isolated probiotic candidate strains being considered for use in probiotic applications. Probiotic candidate strains that have been used historically in food applications, such as starter cultures, may have GRAS status which can serve as the basis of safety for the new dietary ingredient notification. Despite the notification requirement, the exact evidence required to be submitted for safety and efficacy of the new dietary ingredient is not explicitly specified. Following notification, the FDA issues an “acknowledgment of receipt” which is considered a procedural matter, not an attestation of ingredient safety (U.S. Department of Health and Human Services, 2024). This puts the onus of determining product safety on (1) the manufacturer and (2) the consumer (Figure 2). Thus, a thorough understanding of the science behind probiotic strains marketed for oral care is critical.

## 3 Mechanisms of action of oral care probiotics

Many excellent reviews have recently summarized the mechanisms of action of oral probiotics on specific oral diseases including those by Chugh et al. (2020) and Homayouni Rad et al. (2023). In general, these mechanisms fall into one of three categories: (1) competitive species interactions, (2) production of antimicrobials or inhibitory substances, and (3) immune modulation. In the oral cavity, several specific mechanisms of action have been identified and are thoroughly reviewed at the strain level in Section 4 below. In general, the most common mechanism of action of probiotic bacteria in the oral cavity is competitive exclusion (Hibbing et al., 2010). Probiotic bacteria strains directly complete with pathogens for nutrients, resources, and attachment sites. This method is effective against oral pathogens such as *Streptococcus mutans*, which causes cavities (Kreth et al., 2009), and *Tannerella forsythus*, which produces the volatile sulfur compounds associated with halitosis (Han et al., 2023). Additionally, many oral probiotic strains produce antimicrobial metabolites such as antibiotics or hydrogen peroxide that inhibit the growth of oral pathogens (Homayouni Rad et al., 2023). This method of inhibition works well against anaerobic periodontal pathogens such as *Porphyromonas gingivalis* and *Tannerella forsythia*, which cause periodontitis (Hillman et al., 1985). Additionally, recent research has shown that some oral probiotics may be useful in the identification or treatment of oral cancers via immunomodulation pathways that lead to apoptosis of cancer cells or anti-metastasis activity (Mohd Fuad et al., 2023). While these results are promising, it remains critical to identify the mechanism of action at the strain level for probiotic bacteria.

## 4 Probiotics: genera, species, and strains

### 4.1 Streptococcus

**Figure 3:**
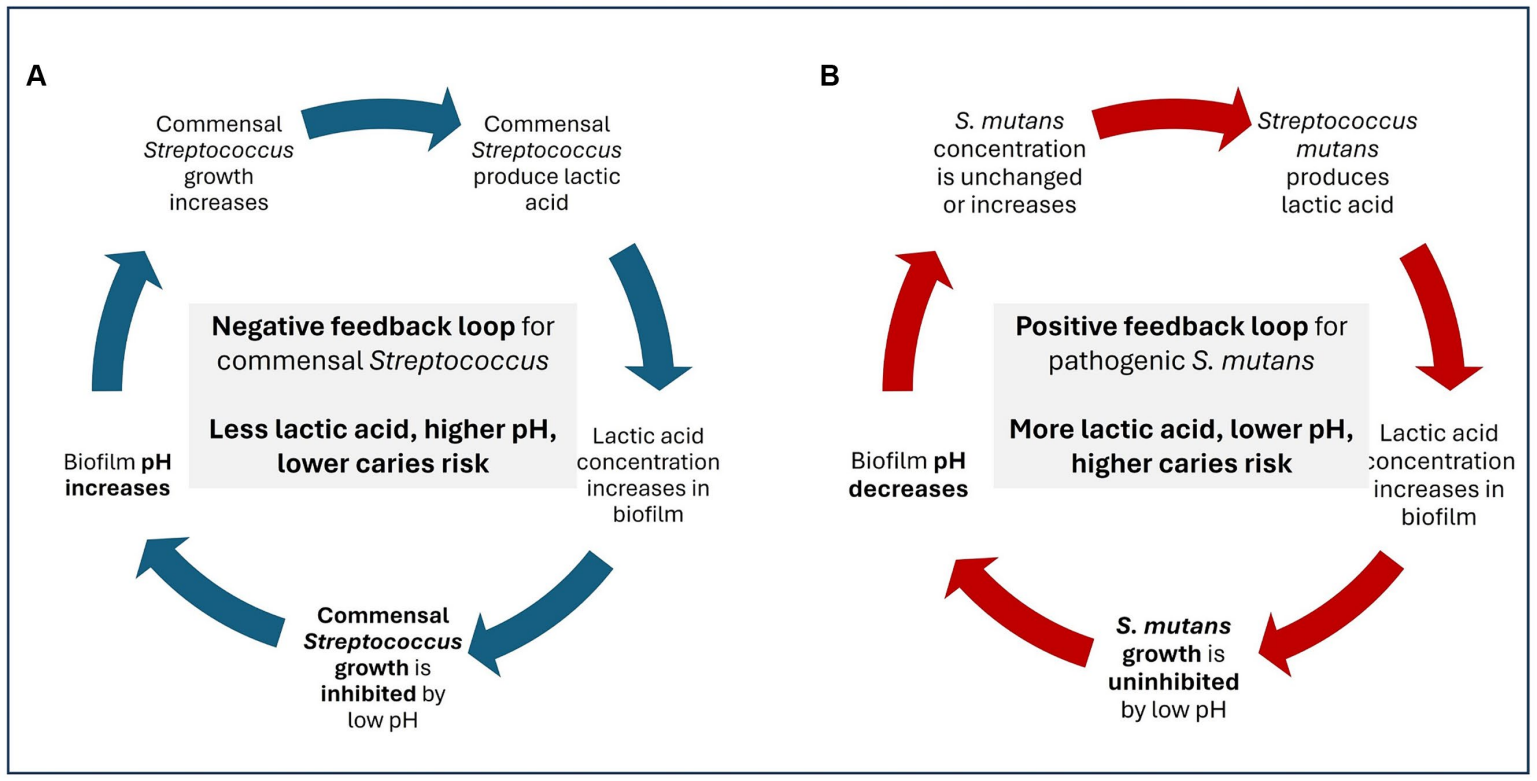
Negative feedback loop **(A)** and positive feedback loop **(B)** of lactic acid production by commensal vs. pathogenic *Streptococcus* spp.

Members of the genus *Streptococcus* are Gram-positive, catalase-negative, lactic acid producing bacteria (Jevitz Patterson, 1996; Du Toit et al., 2014). While a few members of the *Streptococcus* genus are opportunistic pathogens, many streptococci are indigenous commensals in the human microbiome (Abranches et al., 2018; Baty et al., 2022). In the oral cavity, streptococci serve as early colonizers, shaping the oral microbiome and supporting tooth and gum development (Abranches et al., 2018; Sulyanto et al., 2019). Streptococci remain abundant in the oral cavity throughout the transition from childhood to adulthood (Bik et al., 2010; Sulyanto et al., 2019; Ruan et al., 2022) in part due to the production of adhesins. Adhesins produced by streptococci facilitate strong binding teeth and gums. Binding strength is critical for bacterial survival in the oral cavity because saliva flow and food consumption create significant shearing forces capable of displacing bacteria that are more weakly attached (Abranches et al., 2018). Despite their metabolic capacity to produce lactic acid, most commensal oral *Streptococcus* spp. do not contribute to acidogenic tooth decay due to a negative feedback loop in which acid production reduces oral pH which then inhibits the growth of the commensal (Castillo et al., 2000; Figure 3A); this is in contrast to pathogenic oral streptococci which thrive in a low pH environment (Kreth et al., 2009; Figure 3B). Additionally, many oral streptococci contain the arginine deiminase system (ADS) which converts arginine to ammonia and raises local pH (Baty et al., 2022). Together, these factors support commensal streptococci as probiotic candidates.

In the oral cavity, *Streptococcus* spp. supply a health benefit to the host by inhibiting the growth of dental pathogens via metabolic byproducts such as bacteriocins and hydrogen peroxide (Chen et al., 2011; Baty et al., 2022). Oral care probiotic products may contain one or multiple *Streptococcus* spp., but the potential health benefits are strain specific. The most common *Streptococcus* strains found in oral care probiotics are explored in more detail in Table 1.

**Table 1:** Overview of common probiotic candidate species and strains included in oral care probiotics in the United States.

Probiotic strain	Origin of isolation	Native location in human body	Documented probiotic benefit in the oral cavity	Mechanism of action in the oral cavity	Company	Regulatory status	Most common use
*Streptococcus oralis* KJ3	Subgingival plaque of a 32 year-old subject with a clinically healthy periodontium (Hillman et al., 1985)	Teeth, Gingiva	Inhibition of oral pathogens that cause gingivitis and periodontitis, cosmetic whitening (Zahradnik et al., 2009; Hillman et al., 2016)	Hydrogen peroxide production, antimicrobial (Hillman et al., 1985)	ProBiora Health, LLC	Generally Recognized as Safe (food/dietary supplement ingredient)	Oral care probiotic
*Streptococcus uberis* KJ2	Subgingival plaque of a 32 year-old subject with a clinically healthy periodontium (Hillman et al., 1985)	Teeth, Gingiva	Inhibition of oral pathogens involved in gingivitis and periodontitis, cosmetic whitening (Zahradnik et al., 2009; Hillman et al., 2016)	Hydrogen peroxide production, antimicrobial (Hillman et al., 1985)	ProBiora Health, LLC	Generally Recognized as Safe (food/dietary supplement ingredient)	Oral care probiotic
Streptococcus rattus JH145	spontaneous lactate dehydrogenase deficient (LDH-) mutant of *Streptococcus mutans* strain BHT-2 (Hillman et al., 2009)	Teeth	Inhibition and competitive exclusion of oral pathogens that cause caries (Hillman et al., 1987, 2009; Cannon et al., 2013; Hedayati-Hajikand et al., 2015)	LDH- Deficient, Competitive Exclusion (Hillman et al., 2009)	ProBiora Health, LLC	Generally Recognized as Safe (food/dietary supplement ingredient)	Oral care probiotic
*Streptococcus salivarius* BLIS K12	Saliva of a child (Burton et al., 2006)	Dorsum of the tongue and the pharyngeal mucosa	Inhibition of oral pathogens that cause halitosis (Yoo et al., 2020)	Bacteriocin production (lantibiotics salivaricin A and salivaricin B; Hyink Otto et al., 2007)	BLIS, Burst Oral Probiotics, Nature’s Plus, Probium, Naturewise, Probi USA, Mars Wellness, Hyperbiotics, Therabreath	Generally Recognized as Safe (food/dietary supplement ingredient)	Oral care probiotic
*Streptococcus salivarius* BLIS M18	Oral cavity of a healthy adult (Burton et al., 2013)	Dorsum of the tongue and the pharyngeal mucosa	Inhibition of oral pathogens that cause gingivitis, periodontitis, and caries (Burton et al., 2013; Di Pierro et al., 2015; Jansen et al., 2021)	Bacteriocin production (lantibiotics salivaricin A2, salivaricin 9, salivaricin M; Wescombe et al., 2011)	BLIS, Burst Oral Probiotics, Nature’s Plus, Probium, Naturewise, Probi USA, Mars Wellness, Hyperbiotics, Therabreath	Generally Recognized as Safe (food/dietary supplement ingredient)	Oral care probiotic
Limosilactobacillus reuteri DSM 17938	daughter strain of *L. reuteri* ATCC 55730 (originally isolated from human breast milk) with antibiotic resistance plasmids removed (Urbańska and Szajewska, 2014)	Intestine, skin, breast milk; transient member of oral cavity	Conflicting evidence for inhibition of oral pathogens (Vivekananda et al., 2010; Kraft-Bodi et al., 2015; Widyarman et al., 2018; EFSA Panel on Nutrition et al., 2020; Laleman et al., 2020; Schlagenhauf et al., 2020), documented benefits for improved gut health by protecting the mucosal lining (Jiang et al., 2023)	Competitive bacterial interactions (Kraft-Bodi et al., 2015), production of biosurfactants which inhibit pathogen growth (Ciandrini et al., 2016)	BioGaia, SUNSTAR Suisse S.A.	Generally Recognized as Safe (food/dietary supplement ingredient)	Gut probiotic
Limosilactobacillus reuteri ATCC PTA 5289	Oral cavity of a healthy adult woman (Liu et al., 2010)	Intestine, skin, breast milk; transient member of oral cavity	Conflicting evidence for inhibition of oral pathogens (Vivekananda et al., 2010; Kraft-Bodi et al., 2015; Widyarman et al., 2018; EFSA Panel on Nutrition et al., 2020; Laleman et al., 2020; Schlagenhauf et al., 2020), reduces inflammation in the gut (Jiang et al., 2023)	Competitive bacterial interations (Kraft-Bodi et al., 2015), immune signaling (Boisen et al., 2023)	BioGaia, SUNSTAR Suisse S.A.	Generally Recognized as Safe (food/dietary supplement ingredient)	Gut probiotic
Lactobacillus acidophillus (multiple strains)	Multiple, strains rarely defined	Intestine, urinary tract, vagina; transient member of the oral cavity	None, implicated in caries progression (Bunting et al., 1928; Leme et al., 2022)	None	Nuveda Wellness, Great Oral Health, Swanson, Henry Blooms, RENUzORAL	Generally Recognized as Safe (food/dietary supplement ingredient)	Food starter culture; gut probiotic
*Bifidobacterium breve* Bb-03 (B-3)	Human feces (Acharya and Shah, 2002)	Intestine	None	None	Nuveda Wellness, NatureWise	Generally Recognized as Safe (food/dietary supplement ingredient)	Gut probiotic for infants and adults
*Bifidobacterium lactis* Bl-04	human feces (Rodolphe et al., 2009)	Intestine	None	None	Nuveda Wellness, NatureWise	Generally Recognized as Safe (food/dietary supplement ingredient)	Gut probiotic for infants and adults

Details include the original isolation source, mechanism of action, and documented probiotic benefit, if any, in the oral cavity along with relevant literature citations. Companies marketing products that include the listed species and/or strains, the US regulatory status of the strain, and the most common use are also included.

The earliest reported *Streptococcus* strains displaying a probiotic benefit were isolated from the subgingival plaque of a healthy adult subject in 1985 (Hillman et al., 1985). Two strains, *S. oralis* strain KJ3 (previously *S. sanguis* Type II strain KJ3) and *S. uberis* strain KJ2, were shown to have inhibitory effects on the growth of oral pathogens implicated in periodontitis including *Fusobacterium nucleatum*, *Aggregatibacter actinomycetemcomitans*, and *Porphyromonas gingivalis* (previously *Bacteroides gingivalis*; Hillman et al., 1985). The production of hydrogen peroxide by *S. oralis* KJ3 and *S. uberis* KJ2 was found to be the mechanism of action for pathogen growth inhibition. In addition, *S. oralis* strains are early colonizers of the tooth surface, binding strongly to the salivary pellicle (Li et al., 2004; Dorkhan et al., 2013). Colonization of the tooth surface by probiotic *Streptococcus* strains such as *S. oralis* KJ3 and *S. uberis* KJ2 has been shown to shift the microbiome of the oral cavity to a healthier state (Zahradnik et al., 2009). Additionally, the low level of hydrogen peroxide produced by *S. oralis* KJ3 and *S. uberis* KJ2 provide a whitening effect on the tooth enamel (Hillman et al., 2016). Although dental whitening is frequently cited as a cosmetic benefit, whiter teeth have been shown to have a positive social and psychological effect as well (Estay et al., 2020).

*Streptococcus rattus* strain JH145 is another probiotic strain in the *Streptococcus* genus. This bacterium is a spontaneous mutant of a *Streptococcus mutans* strain isolated from a carious lesion in an adult subject (Hillman et al., 2009). In contrast to *S. mutans*, S*. rattus* JH145 is lactate dehydrogenase deficient (LDH-), and thus does not produce lactic acid as part of its metabolism. As an oral care probiotic, *S. rattus* JH145 provides a health benefit through competitive exclusion by consuming the same resources and inhabiting the same ecological niche as *S. mutans* strains (Hillman et al., 1987). This effect has been shown in multiple animal and human clinical studies (Hillman et al., 2009; Cannon et al., 2013, 2019; Hedayati-Hajikand et al., 2015).

*Streptococcus salivarius* strains K12 and M18 also provide probiotic benefits in the oral cavity. These strains were originally isolated from the saliva of a child (*S. salivarius* K12; Burton et al., 2006) and the oral cavity of a healthy adult (*S. salivarius* M18; Burton et al., 2013). It should be noted that *S. salivarius* K12 and M18 are occasionally marketed under the alternative strain identifiers DSM 13084 (strain K12) and DSM 14685 (strain M18), depending on the product. *Streptococcus salivarius* K12 and M18 produce bacteriocins [lantibiotics (McAuliffe et al., 2001; Hyink Otto et al., 2007; Wescombe et al., 2011)] which act in a similar manner to antimicrobials. *Streptococcus salivarius* K12 is frequently cited as an ear, nose, throat, and upper respiratory probiotic (Upton et al., 2001; Zupancic et al., 2017; Bertuccioli et al., 2023) while *S. salivarius* M18 is more often cited as a “true” oral care probiotic inhibiting dental pathogens (Burton et al., 2013; Di Pierro et al., 2015). A combination of the two strains has been shown to reduce immune activation induced by periodontal pathogens (MacDonald et al., 2021), reduce the abundance of periodontal pathogens (Jansen et al., 2021), and reduce the levels of volatile sulfur compounds involved in halitosis (Yoo et al., 2020). In general, these probiotic effects occur due to either (a) inhibition of oral pathogen growth due to bacteriocin production or (b) reducing inflammatory responses by downregulating proinflammatory pathways (MacDonald et al., 2021; Baty et al., 2022). Human clinical trials support the claims that these strains reduce concentrations of caries causing bacteria (Poorni et al., 2022).

*Streptococcus thermophilus,* a member of the *salivarius* subgroup, is occasionally included in products marketed for oral care; however, this species is most frequently used as a starter culture for foods including yogurt and some cheeses (Cui et al., 2016). When this strain is included in probiotic products for oral care, it is found in combination with other bacterial species and is, to the best of this author’s knowledge, never identified to the strain level (How and Yeo, 2021). The lack of strain level information for this species suggests that *S. thermophilus* is not currently a good probiotic candidate for oral care.

### 4.2 *Lactobacillus* (current and reclassified genera)

Until 2020, the genus *Lactobacillus* contained more than 250 bacterial species with distinct phenotypes, genotypes, and ecological niches (Zheng et al., 2020). Today, members of the *Lactobacillus* genus have been reclassified into 25 distinct genera that, despite their new names, continue to comprise a large proportion of the human microbiota (De Angelis and Gobbetti, 2011; Zheng et al., 2020). For simplicity, the name *Lactobacillus* will be used throughout this section with reference made to the reclassified species and strain names where appropriate. As the name suggests, *Lactobacillus* species are lactic acid producing, Gram-positive, catalase negative bacteria that are generally considered aerobic but may be able to tolerate low levels of oxygen (Zotta et al., 2017). Many *Lactobacillus* species are well-known gut probiotics with documented health benefits including immune modulation, competitive exclusion, antimicrobial excretion, and inflammation suppression (for an updated review, see Dempsey and Corr, 2022). However, the safety and efficacy of *Lactobacillus* spp. for probiotic use in the oral cavity is less well understood.

A few *Lactobacillus* spp. are found in the oral cavity of newborn infants (Sulyanto et al., 2019), but these populations are no longer measurable after 1 month or after the cessation of breast feeding (Caufield et al., 2015). *Lactobacillus* spp. are not considered dominant members of the oral cavity, and established populations are often found only in individuals with carious lesions (Caufield et al., 2015). Despite the large body of evidence implicating *Lactobacillus* spp. in the progression of dental caries (Caufield et al., 2015; Ademe et al., 2020; Sounah and Madfa, 2020; Wen et al., 2022), many oral care products on the market contain *Lactobacillus* spp. as probiotics. This may appear to parallel the use of commensal *Streptococcus* spp. as probiotics when *S. mutans* is a well-known cariogenic bacterium as described in Section 4.1. However, *Lactobacillus* spp. in the oral cavity are frequently linked to food products in which *Lactobacillus* strains were used as starter cultures, suggesting they are transient members of the oral microbiota rather than permanent colonizers (Caufield et al., 2015). Research also indicates *Lactobacillus* spp. are not indigenous to the oral cavity (Caufield et al., 2015) and therefore may not be the preferred choice for an oral care probiotic product, especially if their presence is linked to tooth decay. Regardless, due to their popularity, consumer familiarity with the name, and documented health benefits in gut probiotics (Dempsey and Corr, 2022), *Lactobacillus* spp. are readily available for use, cheap, and documented as “safe” (Salminen et al., 1998; Rodríguez-Sánchez et al., 2021), leading to incorporation into many marketed oral care probiotic products. The most common *Lactobacillus* (or former *Lactobacillus*) species and strains used in oral care probiotics are described in more detail in Table 1.

*Lactiplantibacillus plantarum* (formerly *Lactobacillus plantarum*) strain 299 (or DSM 6595) and *Lactiplantibacillus plantarum* strain 299v (or DSM 9843) were originally isolated from the mucosa of a healthy human intestine (Molin et al., 1993). These two strains are well studied as probiotics for the gut (Nordström et al., 2021). Potential probiotic benefits of these strains in the oral cavity have also been explored with studies showing that *L. plantarum* 299v can co-aggregate with *S. mutans* in carious lesions (Twetman et al., 2009) and can inhibit biofilm formation of clinical isolates of *S. mutans* (Söderling et al., 2011). However, additional research shows that *L. plantarum* 299v produces significantly more lactic acid than other *L. plantarum* strains (Haukioja et al., 2008; Keller and Twetman, 2012) which suggests that the acidogenicity of the strain needs to be considered prior to promoting its use as an oral care probiotic. Another *L. plantarum* strain, HEAL19 (or DSM 15313), has recently been incorporated into oral care probiotic products (Durrell, 2021). However, PubMed has only indexed a total of seven peer-reviewed research papers citing this bacterial strain and none of them support the use of this strain for oral health. Additional strains of *L. plantarum* have been isolated, and a few have been studied for oral health benefits [such as strains L-137 (Schlagenhauf and Jockel-Schneider, 2021), DSM 32131 (Volgenant et al., 2022), NC8 (Khalaf et al., 2016), and 44048 (Khalaf et al., 2016)]. The results suggest that strains of *L. plantarum* can survive in the oral cavity, but their inhibition of oral pathogens is strain and pH dependent. In conclusion, not all strains of *L. plantarum* behave similarly in the oral cavity and more research is needed to confirm a probiotic benefit.

*Lacticaseibacillus paracasei* strains including 8,700:2 (or DSM 13434), Lpc-37, ET-22, SD1, and adp-1 are also used in oral care probiotics (How and Yeo, 2021). *Lacticaseibacillus paracasei* strains ET-22 and SD1 do have some support as oral care probiotics in the literature; these strains have been shown to suppress the formation of caries by inhibiting the formation of biofilm (Guo et al., 2023; Zhao et al., 2023) or reducing the concentration of *S. mutans* in the oral cavity (Teanpaisan et al., 2015). However, limited placebo-controlled, double-blinded clinical studies are available and most of the research on *L. paracasei* strains remains in gut health.

*Limosilactobacillus reuteri* strains, especially DSM 17938 and ATCC PTA 5289, are also frequently listed as probiotics for oral care. *Limosilactobacillus reuteri* DSM 17938 is a daughter strain of *L. reuteri* ATCC 55730 that was originally isolated from human breast milk (Urbańska and Szajewska, 2014). *Limosilactobacillus reuteri* ATCC 55730 contained potentially transferable antibiotic resistance plasmids; thus, the daughter strain DSM 17938, in which the antibiotic resistance plasmids were removed, is used in probiotic products (Urbańska and Szajewska, 2014). *Limosilactobacillus reuteri* ATCC PTA 5289 was isolated from the oral cavity of a healthy adult woman (Liu et al., 2010). Oral outcomes for these strains are mixed; some studies suggest that they improve gingival health (Schlagenhauf et al., 2020) and reduce the concentration of oral pathogens (Kraft-Bodi et al., 2015; Widyarman et al., 2018), however, research also shows that indices such as bleeding on probing may not be improved (Kraft-Bodi et al., 2015) and oral pathogen levels are not always reduced following use (Vivekananda et al., 2010; Laleman et al., 2020). Interestingly, in 2020, the European Food Safety Authority made a definitive statement that current research on *L. reuteri* strains DSM 17938 and ATCC PTA 5289 is insufficient to determine if they provide a health benefit for the gums (EFSA Panel on Nutrition et al., 2020). Additional research is warranted to determine the efficacy of these strains as oral care probiotics.

The strains detailed above are not exhaustive of the current and former *Lactobacillus* species included in oral care probiotics; see How and Yeo (2021) for a thorough list. *Lactobacillus* spp. remain the dominant group of bacteria included in probiotic products, regardless of the body area in which the health benefit should occur. This has led to an abundance of marketed products that do not contain enough information for the consumer to make an informed decision and is exacerbated by a lack of strain level information on probiotic product labels (Weese and Martin, 2011). Together, these factors lead to the scenario described in Section 2; individuals take a probiotic product under the assumption that it will provide a health benefit in the mouth when it may actually be contributing to oral health problems. One such species is *Lactobacillus lus*, which is often included in probiotics for oral care with no strain identifier (How and Yeo, 2021). As noted previously, strain level information is critical for probiotics as the same bacterial species may vary widely in gene content at the strain level. *Lactobacillus acidophilus* (formerly *Bacillus acidophilus*) was one of the first bacteria identified in the progression of dental caries (Bunting et al., 1928; Johnston et al., 1933). More recent research suggests *L. acidophilus* is frequently found in carious lesions (Leme et al., 2022) and may form dual species biofilms with *S. mutans* (Mei et al., 2013). Although *L. acidophilus* ATCC 4356 was able to induce downregulation of glucan production in co-cultured *S. mutans* (which may reduce biofilm formation), the *Lactobacillus* strain was still able to incorporate into the oral biofilm (Lee and Kim, 2014) where it can contribute to acid production. Together, these results suggest that *L. acidophilus* (or any bacterium at the species level only) is not an appropriate candidate for an oral care probiotic, and yet it is still found in many probiotic products targeted for oral health.

### 4.3 Bifidobacterium

Members of the genus *Bifidobacterium* are Gram-positive anaerobes that are predominantly found in the human gastrointestinal tract. Although the *Bifidobacterium* genus contains over 90 species (Chen et al., 2021), few are found as indigenous commensals in the oral cavity. For example, although *B. dentium* and *B. longum* have been isolated from the mouth (Modesto, 2018), these species are often found associated with carious lesions (Dige et al., 2014; Manome et al., 2019). In the gut, *Bifidobacterium* spp. are used as probiotics. Milk-based formula may include *Bifidobacterium* spp. as they have been shown to reduce the risk of gastroenteritis and stimulate the immune system in infants (Lemoine et al., 2023). Additionally, some strains of *Bifidobacterium* are used in psychological health and may help reduce stress and anxiety (Chen et al., 2021).

In the oral cavity, the probiotic benefits of *Bifidobacterium* are not well defined. A recent meta-analysis of the role of *Bifidobacterium* spp. in the oral cavity concluded that limited evidence of a health benefit exists, and additional research is required (Jayachandra et al., 2023). A similar comprehensive review found that *Bifidobacterium* spp. research in the oral cavity is conflicting, with some studies showing positive reductions in caries causing bacteria and others showing increased acidity and carcinogenicity when *Bifidobacterium* strains are introduced (Homayouni Rad et al., 2023). Specific *Bifidobacterium* strains that are included in common oral care probiotics include *B. breve* strain Bb-03 (or B-3) and *B. lactis* Bl-04 (How and Yeo, 2021; Table 1). Based on a PubMed and Google Scholar search of both strains, no research exists showing a health effect in the oral cavity following use. However, when *Bifidobacterium* strains including *B. lactis* Bb-12 and *B. bifidum* ATCC 29521 were consumed in food products such as ice cream or yogurt, significant reductions in *S. mutans* were found (Homayouni Rad et al., 2023). However, these *Bifidobacterium* strains were often combined with *Lactobacillus* spp. so the probiotic effect cannot be directly linked to *Bifidobacterium* spp. alone. As with *Lactobacillus* spp. many oral care probiotics on the market containing *Bifidobacterium* do not include the strain identifiers necessary for consumer transparency and confidence (How and Yeo, 2021). Together, these results suggest that *Bifidobacterium* spp. are not well supported as oral probiotic strains.

### 4.4 Bacillus

Members of the genus *Bacillus* are gram-positive, spore forming bacteria that can survive in either aerobic or facultative anaerobic environments. As spore formers, *Bacillus* spp. are resilient to temperature fluctuations, desiccation, and many disinfectants (Turnbull, 1996). This can make *Bacillus* spp. difficult to kill, and members of the genus are often implicated in food spoilage. In the human body, *Bacillus* are found in the gut (Lee et al., 2019). Although they are often used as probiotics for crops and livestock (Leistikow et al., 2022), their use as probiotics for human health is less common due to the propensity of *Bacillus* spp. to transfer antibiotic resistance genes and produce toxic biproducts (Lee et al., 2019).

Only one *Bacillus* species was found in marketed oral care probiotics: *Bacillus coagulans* strain Unique IS2 (or ProDura; How and Yeo, 2021). A recent placebo controlled, double-blind clinical trial found that following 14 days of *B. coagulans* Unique IS2 application, oral levels of *S. mutans* and *Lactobacillus* spp. were significantly reduced (Ratna Sudha et al., 2020). However, the mechanism of action was not identified. Additional evidence of efficacy is needed for this oral care probiotic candidate, and caution should be used for any potential *Bacillus* probiotic based on the factors noted above.

## 5 Prebiotics and postbiotics

### 5.1 Prebiotics

Like probiotics, prebiotics were originally conceived for gut health. Prebiotics were first defined as a, “non-digestible food ingredient that beneficially affects the host by selectively stimulating the growth and/or activity of one or a limited number of bacteria in the colon, and thus improves host health (Gibson and Roberfroid, 1995).” Today, the official definition of prebiotics has expanded in scope to include, “a substrate that is selectively utilized by host microorganisms conferring a health benefit (Gibson et al., 2017),” which includes ingredients that stimulate growth and/or activity of bacteria in the oral cavity. It is important to note that probiotic bacteria, if well-selected for the body area of interest, do not require a prebiotic to confer a health benefit. However, prebiotics may encourage growth of probiotics strains providing an additional benefit.

In the oral cavity, a variety of prebiotics have been investigated. These include some sugars, sugar alcohols, oligosaccharides (complex sugars), amino acids, and nitrogen species (Luo et al., 2024). Prebiotics must be carefully selected to encourage the growth of probiotic strains without stimulating the growth of oral pathogens. Examples of well-researched prebiotics for oral care include xylitol (a sugar alcohol), arginine (an amino acid), and urea (a nitrogen species). Multiple studies have shown xylitol reduces levels of *S. mutans* in plaque and saliva (Milgrom et al., 2006, 2009; ALHumaid and Bamashmous, 2022) primarily because *S. mutans* strains cannot ferment xylitol (Nayak et al., 2014). This results in an increase in oral pH. Arginine is a relatively new prebiotic shown to neutralize oral pH (Nascimento, 2018) as a precursor to nitric oxide. The arginine deiminase pathway of many commensal oral bacteria (including *S. oralis* and *S. rattus*) produces alkali compounds which inhibits the formation of acidic plaque (Zheng et al., 2017; Nascimento, 2018). Urea works similarly to arginine in the oral cavity, as microbial metabolism converts urea to ammonia, raising the oral pH (Mora and Arioli, 2014).

Although prebiotics for oral health appear promising, additional research is needed to verify if these compounds support the growth of probiotic bacterial species in the mouth. Additionally, most prebiotics are targeted to increase oral pH, which plays a role in caries development and prevention. However, little is known about prebiotics to support other oral health conditions such as gingivitis and halitosis.

### 5.2 Postbiotics

Postbiotics are the newest component of microbially derived products that confer a health benefit. Defined in 2019 as, “a preparation of inanimate microorganisms and/or their components that confers a health benefit on the host,” postbiotics are compounds that do not require, but must be prepared from, live microorganisms (Salminen et al., 2021). Postbiotics can include any portion of a microbial cell such as the cell wall or cytoplasm or a microbial metabolite. Most often, postbiotics are compounds that are released by live microorganisms that confer a health benefit, such as hydrogen peroxide and bacteriocins produced by members of the *Streptococcus* genus (Homayouni Rad et al., 2023). Postbiotic research is still in its infancy, but research has shown that the fermentation products of probiotic strains can inhibit the growth of oral pathogens in the absence of the live probiotic strain (Lin et al., 2022). These results are encouraging and provide an alternative avenue for producing the health benefit of probiotic strains without the manufacturing and storage constraints of maintaining a live microorganism. Additionally, the inherent risk of consuming live microbial products is significantly reduced by supplying only the metabolite or cellular component rather than living cells.

## 6 Discussion and considerations

Oral health disparity in the United States remains a serious challenge (National Institute of Dental and Craniofacial Research, 2021). Caries, gingivitis, and periodontitis continue to affect a significant portion of the population despite targeted treatment efforts (National Institute of Dental and Craniofacial Research, 2021). Alternative therapies, such as the probiotics described here, are gaining traction. The North American oral probiotic market alone was estimated at a value of more than 100 million USD in 2022 with an expected growth of nearly 10% by 2030 (Grand View Research, 2022). Consumer trends are driving the oral probiotic market, with preferences for more holistic and natural solutions to oral health problems (Grand View Research, 2022). Unfortunately, this has led to an influx of probiotic products marketed for oral care that contain bacterial strains with a history of safe use but no documented oral health benefits. These strains are the easiest to acquire, manufacture, and include in a product despite their lack of efficacy in the oral cavity. This increases consumer confusion regarding strain choice, safety, and potential health benefits of probiotic products.

Although many probiotic strains have been shown to be safe and effective for a variety of oral health conditions, information provided from probiotic companies is not always clear. Consumers are more likely to place trust in information provided by probiotic scientists than from news media or online content; however, science-based information, such as peer-reviewed journal articles, are often inaccessible to consumers (Vijaykumar et al., 2022). Probiotic recommendations for oral care may be more widely accepted from dental clinicians, but clinicians themselves often receive conflicting information and evidence about probiotic products. To help clarify misinformation, the U.S. Department of Health and Human Services prepared a probiotic fact sheet specifically targeted for health professionals; however, probiotics for oral care are not included (National Institutes of Health Office of Dietary Supplements, 2023). This oversight leaves health professionals and consumers to source information themselves from either (a) product labels or (b) the company manufacturing the probiotic products for sale. Due to the presumption of safety rule for probiotic products in the U.S. (meaning products are assumed safe until proven otherwise), consumers are responsible for evaluating the marketing and science messaging probiotic companies provide to determine safety and efficacy. This model is inefficient and may lead to the consumption of probiotic products with no health benefit at best or serious health complications at worst. So how can consumers and dental professionals make informed choices about probiotic products for oral care? A thorough review of the following information is suggested:

1. Does the product label contain the probiotic strain name (s) and active ingredient dosage(s)? Probiotics included in the product should be identified beyond the species level and the specific strain should be easily identifiable.
2. Is there a history of safety and efficacy easily available for the listed probiotic strain? This information should be readily provided by the manufacturer and should include peer-reviewed research articles and summaries accessible to the general public that demonstrate a health benefit in the oral cavity. Clinicians and consumers should also be aware a “familiar” probiotic name (i.e., a species or strain included in multiple products) does not guarantee the probiotic is effective.
3. Does the probiotic strain provide a health benefit for the specific oral condition of concern, such as reducing the bacterial pathogens that contribute to cavity formation or whitening teeth? As a reminder, many common bacterial strains included in oral care probiotics have a history of safe use, usually in food products like yogurt and milk, which makes them simple and quick for manufacturers to include in products to meet consumer demand but does not guarantee a health benefit.
4. Does the product claim to treat, prevent, or cure any disease? These types of claims are not allowed on dietary supplements containing probiotics, as probiotics are not classified as drugs. Instead, the specific health benefit should be described, such as reducing oral pathogens or inhibiting the bacteria that cause bad breath.

As described in detail in this review, probiotic benefits are highly strain specific. Many bacterial species and strains used in probiotic products marketed for oral care have no documented benefit in the oral cavity and are used based on a history of safe use, rather than efficacious use. Of the probiotic genera for oral health reviewed here, *Streptococcus* spp. have the strongest support for health benefits in the oral cavity including reducing levels of oral pathogens, reducing the incidence of caries, reducing levels of halitosis causing bacteria, improving oral pH, and whitening teeth. Members of the genus *Lactobacillus* (or former members of *Lactobacillus*) have mixed support, with some research suggesting they may reduce levels of oral pathogens and inflammation but other studies suggesting the evidence of a health benefit is insufficient. *Lactobacillus* spp. are frequently included in oral care probiotics at the species level only due to their history of safe use as food starter cultures; however, safety does not guarantee efficacy, especially in the oral cavity. Both *Bifidobacterium* and *Bacillus* have little to no documented support for use as oral care probiotics with no specific oral health benefit directly linked to individual strains from these genera.

While the steps listed for consideration above require effort on the part of the consumer and/or clinician, they are critical to ensure health and safety. Additional research is still needed before probiotics are widely accepted as a part of proactive dentistry. Although reported side-effects of probiotic use are minimal to non-existent, there is not yet enough information to provide probiotic dosages for the general population. Regulatory roadblocks in the United States also impact the widespread use of probiotics for oral healthcare. No probiotic strain to date has been approved to treat, prevent, or cure oral health disease. These factors prevent most probiotic products from being covered by insurance providers, increasing the up-front cost to consumers. Future studies investigating health, social, and economic outcomes of long-term probiotic use for proactive dentistry would be beneficial to increase support and widespread adoption.

## Author contributions

RB: Writing – review & editing, Writing – original draft, Visualization, Investigation, Data curation, Conceptualization.
